# Internal Hernia With Enteroenteric Fistula After Roux-en-Y Gastric Bypass in an Adult Female

**DOI:** 10.7759/cureus.102595

**Published:** 2026-01-29

**Authors:** Michael W Alchaer, Harrison Gorran, Stephanie Gonzalez, Koji Honda, Thomas A Abbruzzese

**Affiliations:** 1 General Surgery, HCA Healthcare/University of South Florida (USF) Morsani College of Medicine Graduate Medical Education (GME) HCA Florida Brandon Hospital, Brandon, USA; 2 Department of Medicine, Alabama College of Osteopathic Medicine, Dothan, USA

**Keywords:** enteroenteric fistula, internal hernia, robotic surgery, roux-en-y gastric bypass, small-bowel obstruction

## Abstract

Internal hernia is a recognized late complication of Roux-en-Y gastric bypass (RYGB), often occurring through mesenteric defects at the jejunojejunostomy (JJ) or Petersen’s space. While most hernias result from anatomical defects, the development of an enteroenteric fistula between small-bowel limbs is exceedingly rare. Such fistulae can mimic adhesive bands or internal hernias, creating diagnostic challenges and potential delays in management.

A 39-year-old woman with morbid obesity, RYGB eight years prior, and gastric pouch revision three years prior presented with severe abdominal pain, nausea, and vomiting. CT imaging showed small-bowel obstruction (SBO) with a transition point near the JJ. Repeat CT with oral contrast confirmed persistent obstruction. Emergent robotic laparoscopy identified an internal hernia caused by a short fistulous tract connecting the biliopancreatic limb to the common channel. The fistula was stapled, excised, and sent for histopathologic confirmation, which demonstrated an enteroenteric fistula with focal acute inflammation. The patient recovered uneventfully, resumed bowel function by postoperative day three, and was discharged on postoperative day four, tolerating a regular diet.

Enteroenteric fistula formation after RYGB is extremely rare, with few documented cases in the literature. These abnormal tracts can alter bowel mechanics and mimic internal hernias on imaging, making preoperative diagnosis difficult. CT findings may suggest obstruction, but rarely demonstrate the actual communication. Early operative exploration remains crucial when obstruction persists despite non-diagnostic imaging. Robotic-assisted laparoscopy offers superior visualization and precise dissection, enabling safe fistula excision and rapid recovery.

Internal hernia secondary to an enteroenteric fistula represents a rare and underrecognized cause of SBO after RYGB. Maintaining a high index of suspicion and employing minimally invasive techniques are key to ensuring timely diagnosis and excellent postoperative outcomes.

## Introduction

Internal hernia is a well-recognized late complication of Roux-en-Y gastric bypass (RYGB), typically occurring through mesenteric defects at the jejunojejunostomy (JJ) or Petersen’s space [1,2]. Closure of these defects has been shown to reduce postoperative herniation, yet small-bowel obstruction (SBO) remains one of the most frequent delayed presentations following RYGB. In contrast, enteroenteric fistula formation between small-bowel limbs is exceedingly rare and represents an underreported etiology of obstruction [3-6]. These abnormal tracts can create inter-loop adhesions, distort normal anatomy, and mimic adhesive or internal hernia patterns, leading to diagnostic challenges [5,6]. We present a unique case of an internal hernia secondary to an enteroenteric fistula between the biliopancreatic limb and the common channel, successfully managed with a robotic-assisted approach.

## Case presentation

A 39-year-old woman with morbid obesity, status post-RYGB eight years prior and gastric pouch revision three years prior, presented with one day of sharp central and upper abdominal pain associated with nausea and multiple episodes of vomiting. She was undergoing medical weight loss therapy with semaglutide.

On presentation, her vital signs were within normal limits. Physical examination revealed an obese patient with a soft, nondistended abdomen and tenderness localized to the left hemiabdomen without rebound tenderness or guarding. Laboratory evaluation, including complete blood count and metabolic panel, was within normal ranges.

Computed tomography (CT) imaging of the abdomen and pelvis demonstrated dilated small-bowel loops measuring up to 6 cm with decompressed distal bowel, a transition point near the jejunojejunal (JJ) anastomosis, and mild mesenteric stranding. A repeat CT scan with oral contrast showed persistent SBO without distal contrast passage.

Given concern for a closed-loop obstruction, the patient was taken emergently to the operating room for robotic diagnostic laparoscopy. Abdominal access was obtained via Optiview entry at Palmer’s point, with placement of two additional 8-mm robotic trocars. The small bowel was systematically examined from the terminal ileum proximally. At the level of the JJ anastomosis, a dense inter-loop adhesion with incarcerated small bowel was identified. Reduction of the herniated bowel restored distal bowel filling.

**Figure 1:**
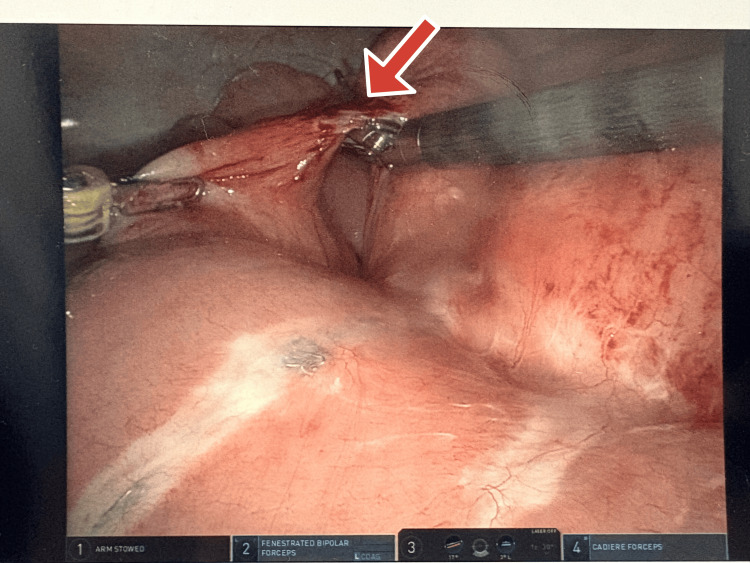
Intraoperative view of the internal hernia with incarcerated small bowel. Arrow pointing at an internal hernia.

**Figure 2:**
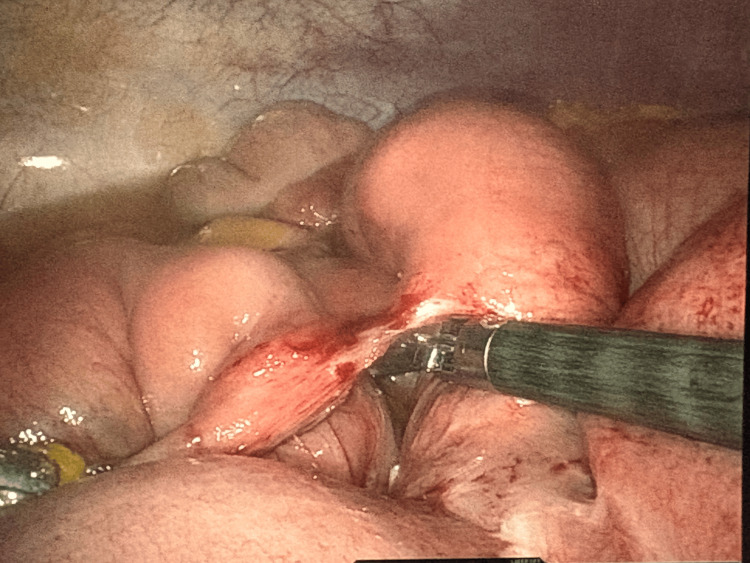
Reduction of the hernia showing viable small bowel loops.

Further inspection revealed chylous ascites and active fluid oozing from a short tract, consistent with an enteroenteric fistula between the biliopancreatic limb and the common channel (Figure 1 and Figure 2). The left upper quadrant port was upsized to facilitate the use of a robotic stapler. The fistulous tract was stapled, excised, and sent for histopathologic evaluation. A Jackson-Pratt drain was placed in the left lower quadrant due to significant ascites.

Pathologic examination confirmed an enteroenteric fistula with focal acute inflammation. The patient recovered without complications, regained bowel function by postoperative day three, and was discharged home in stable condition on postoperative day four while tolerating a regular diet.

## Discussion

Internal hernia is a well-known late complication of RYGB, most often occurring through mesenteric defects at the JJ or Petersen’s space. Closing these defects during the primary operation significantly decreases the risk of herniation [1,2]. In contrast, enteroenteric fistula formation between small-bowel limbs is exceedingly rare and represents an unusual cause of SBO. These tracts can create inter-loop adhesions, distort anatomy, and mimic adhesive or internal hernias, leading to diagnostic challenges [5,6].

Most reported post-bypass fistulae involve the gastric pouch or gastrojejunal anastomosis rather than the jejunojejunal configuration seen here [3-7]. The proposed mechanisms include chronic inflammation, localized ischemia, staple-line failure, or prior revisional surgery [3,4]. In this patient, previous pouch revision likely contributed to localized inflammation and subsequent fistula formation between the biliopancreatic limb and the common channel.

CT imaging remains the main diagnostic tool for SBO after RYGB, but short fistulous tracts are often undetectable even with oral contrast [2,8-11]. Persistent obstruction despite non-diagnostic imaging should prompt early surgical exploration [4,8]. Minimally invasive approaches, particularly robotic surgery, enable comprehensive inspection of mesenteric defects and precise management of rare findings such as fistulae [1].

This case emphasizes that an enteroenteric fistula should remain in the differential diagnosis for obstruction following RYGB [3-6]. Prompt recognition and minimally invasive repair can achieve excellent outcomes while preserving bowel integrity [1,9,12].

## Conclusions

Internal hernia secondary to an enteroenteric fistula is an exceptionally rare cause of SBO after RYGB. Surgeons should maintain a high index of suspicion in post-bariatric patients presenting with unexplained obstruction, even when imaging is inconclusive. Early recognition and minimally invasive repair, particularly with robotic assistance, can lead to excellent outcomes while preserving bowel integrity.
